# Density-Based Topology-Optimized 3D-Printed Fixtures for Cyclic Mechanical Testing of Lattice Structures

**DOI:** 10.3390/polym17182468

**Published:** 2025-09-12

**Authors:** Josué Castro, Rodrigo Valle, Jorge Leiva, Angelo Oñate, Enrico Saggionetto, Anne Mertens, Víctor Tuninetti

**Affiliations:** 1 Department of Mechanical Engineering, Universidad de La Frontera, Temuco 4811230, Chile; j.castro09@ufromail.cl; 2 Facultad de Arquitectura, Construcción y Medio Ambiente, Universidad Autonoma de Chile, Talca 3460000, Chile; rodrigo.valle@uautonoma.cl; 3 Composite Materials Laboratory, Department of Mechanical Engineering, Universidad de La Frontera, Temuco 4780000, Chile; 4 Department of Materials Engineering (DIMAT), Faculty of Engineering, Universidad de Concepción, Concepción 4070138, Chile; aonates@udec.cl; 5 Metallic Materials for Additive Manufacturing (MMS), A&M Department, University of Liège, Quartier Polytech 1 Allée de la Découverte 13 (B52), 4000 Liège, Belgium; enrico.saggionetto@uliege.be (E.S.); anne.mertens@uliege.be (A.M.)

**Keywords:** topology optimization, 3D printed fixtures, cyclic mechanical testing, lattice structures, additive manufacturing

## Abstract

The reliable experimental characterization of architected lattice materials under cyclic loading requires accurate fixture systems that ensure proper load transfer without introducing parasitic effects. This study presents the design and validation of testing fixtures optimized using density-based topological optimization techniques for performing cyclic load tests on lattice structures. The supports were manufactured with PLA filaments and evaluated using finite element simulation and experimental testing. The results show that the final design achieved a safety factor of 4.25, significantly improving on the initial value of 2.08. Likewise, the optimized supports showed reduced deformations by around 80% compared to the machine clamps, ensuring rigid and reliable stress transfer. In particular, while the metal structure of the test system showed deformations of several millimeters, the optimized PLA supports recorded displacements around 0.73 mm, confirming that they remain virtually rigid and ensure correct transmission of forces to the Kelvin-type structure. These findings confirm the viability of using PLA as an alternative to conventional metal devices in fixtures for mechanical testing of lattice materials.

## 1. Introduction

The development of lattice structures fabricated by additive manufacturing (AM) has opened up new possibilities for the design of advanced mechanical components, especially in applications that demand low weight, high energy dissipation, and controlled responses to dynamic loads. Since, improving the mechanical performance in advanced structures is generally achieved by extending the geometric complexity to a level that is impossible to fabricate with traditional manufacturing techniques [1]. In this context, the demand for improved properties in materials can be addressed by developing materials with optimized geometry [2]. The ability of AM to handle complex geometries with ease has opened many avenues in topological optimization, enabling lightweight structural design without sacrificing mechanical performance [3,4,5].

In this way, topological optimization presents itself as a highly promising tool for structural design, allowing the strategic addition of material as a function of the structural load path [6]. The literature demonstrates that this methodology is highly versatile and not only allows flexibility in design but also maximizes the strength-to-weight ratio of structures [7,8,9]. Topological structure frequently delivers the design of geometrically complex structures and 3D printing may represent the only viable solution for their fabrication [10]. Numerous successful applications of topological optimization through 3D printing have been achieved, from feature control to the design of stable auxetic metamaterials [11] where the local volume fraction and a skeleton-based length scale function are combined to regulate the material distribution and minimum geometric size ensuring connectivity between cells. There even is research where this strategy is applied to topology optimization for fluid flow paths [12]. The study demonstrates that the combination of advanced numerical simulation, local control rules, and topological optimization algorithms allows for designing flow channels adaptable to complex geometries and more efficient engineering applications. Similarly, there has been implementation of topological optimization techniques in the robotic area in the development of a structurally optimized mobile robotic system to reduce structural weight while maintaining mechanical integrity [13]. Also in the prosthetic area, in [14] a topological optimization approach is used to design a new wrist brace with improved mechanical performance and greater comfort for the user, adapting precisely to the patient’s anatomy, minimizing material usage and weight. In [15] a metamaterial is implemented to the design of a non-pneumatic tire with the aim of increasing compressive strength and flex fatigue life. An optimization was performed for three types of topologies and combining three types of materials: polylactic acid (PLA), thermoplastic polyurethane (TPU), and void.

Moreover, given the potential that topological optimization offers to computational design, it is able to produce innovative and disruptive structural configurations. In addition, from the pioneering work of Bendsoe and Kikuchi [16], the literature demonstrates the development of other methods, such as the level set method (LSM) [17,18,19], the solid isotropic material with penalty (SIMP) [20,21,22], the evolutionary structural optimization (ESO) method [23,24,25], the moving morphable component (MMC) [26,27] and empty morphable component (MMV) methods [28,29,30], the geometry projection method, and feature-based approaches [31]. Similarly, deep learning (DL)-based approaches [32] can address challenges mainly using 2D shapes and also on low-resolution 3D geometries. These topological optimization methods have been successfully applied in various industries, such as aerospace [33,34], automotive [35,36,37], energy efficiency [38,39], electronics [40,41], and biomedical [42,43,44]. Thus, the literature is extensive in studies combining AM and topological optimization, proving to be a powerful tool for the design and fabrication of new devices, significantly improving mechanical performance and considerably reducing weight of structures [45,46,47,48,49].

However, despite the steady increase in studies focused on the characterization of lattice structures by mechanical testing, the design of the fixtures used in such tests has received little attention in the literature. In many cases, the fixtures are designed without numerical or mechanical validation, which can introduce parasitic stiffnesses, undesired irregular deformations in the specimen [50,51]. Similarly, in [52] it has been shown that the geometry of manufacturing tools and process parameters can significantly affect mechanical responses, such as surface waviness and natural frequency in metal structures. This highlights the importance of carefully considering manufacturing geometry when designing experimental setups for mechanical testing. Some research has begun to address these limitations using approaches, such as 3D printing of auxiliary devices in engineering polymers [53,54] combined with finite element simulations (FEM), to verify their structural strength. Despite significant advances in the design and optimization of cellular structures for engineering applications, the literature presents limitations regarding the design of clamping devices for mechanical cyclic load testing. Most studies have focused on the structural response of lattices and their properties under different load conditions [55,56,57,58,59], leaving the development of optimized supports that ensure rigid and reliable stress transmission during testing in the background. Consequently, there is no established methodological framework that integrates topological optimization into the design of fixtures for this type of testing. Therefore, this work addresses this gap through the design and topological optimization of supports made of PLA, specifically designed for cyclic load testing on Kelvin-type structures, proposing an innovative and replicable solution for future testing devices. The novelty of this research lies in a fixture design that advances the mechanical characterization of reticular materials. This is achieved through three key features: an optimized geometry with progressive stiffeners to maximize the safety factor with minimal mass increase; a polymer construction proven to be a rigid and reliable alternative to metal for load transfer; and a cyclic stress transmission mechanism that ensures test repeatability by reducing support deformation.

## 2. Materials and Methods

### 2.1. Test Protocol Under Cyclic Loads

**Figure 1:**
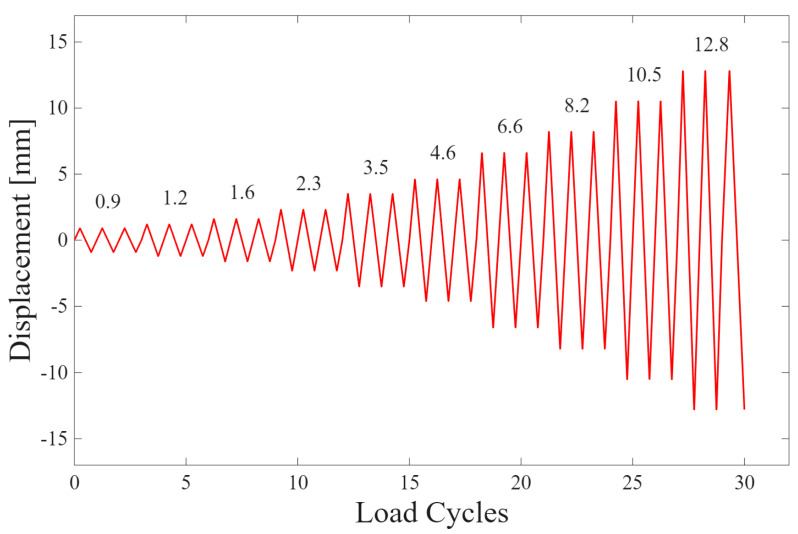
Cyclic loading protocol for mechanical testing.

In this research an incremental loading protocol is applied, consisting of three cycles per displacement level, following the approach used in Steel Plate Shear Yielding Dampers (SPSYD) shock absorber testing. The displacement values were proportionally adapted following the methodology used in [60], where a maximum displacement of 27.5 [mm] was applied on a specimen with effective width *H* = 140 [mm]. In this study, the protocol was scaled proportionally as a function of the tested specimen width of *H* = 65.25 [mm], keeping the relative strain ratio constant. The resulting displacements (Figure 1) were applied at a constant rate of 0.5 [mm/s]. All tests were performed on an Instron 8801 servo-hydraulic testing machine.

### 2.2. Design of Testing Support

**Figure 2:**
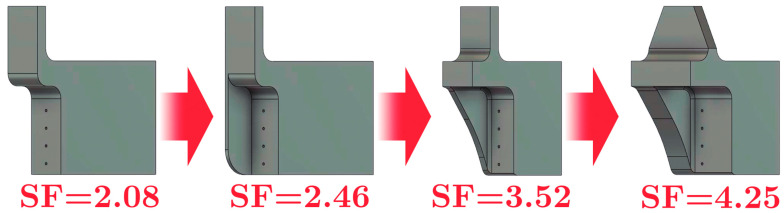
Evolution of the design of the support device for mechanical testing through four iterative stages.

The support was designed to ensure uniform axial load transfer during cyclic quasistatic tests applied to Kelvin-type reticular specimens fabricated by laser powder bed fusion (LPBF). A modular architecture was used, compatible with the fixture plates of an Instron 8801 testing machine, ensuring both axial alignment and geometric stability during the application of alternating loads. The support was modeled in CAD to be manufactured using 3D printing by fused filament deposition (FFF) with PLA filament. From a functional point of view, its modular design allows direct coupling with the jaws of the Instron 8801 testing machine, ensuring proper axial alignment and minimizing eccentricity in the application of load. In previous research, the mechanical properties of PLA have been characterized by Rilling et al. [61] who quantified the effect of fill density and print orientation on the modulus of elasticity and ultimate tensile strength in parts printed in 3D using FFF. Characterization reveals that solid PLA exhibits slightly anisotropic behavior; in particular, the ultimate strength is dependent on the print orientation. Parts loaded parallel to the printed layers are stronger than those loaded perpendicularly against the weaker layer interfaces. Likewise, the material’s stiffness shows greater rigidity in the in-plane direction compared to the more flexible build direction. Poisson’s ratio, which describes transverse contraction, was shown to differ between the primary orientations. For modeling PLA fixtures, these findings could mandate the use of an anisotropic or orthotropic material model in finite element analysis instead of a simple isotropic one. However, these differences for solid material accurately characterized without perimeter tracks or printed envelope show negligible variations. For very accurate simulation, assigning distinct direction-dependent properties for stiffness, strength, and Poisson’s ratio that correspond to the fixture’s intended print orientation may ensure high model accuracy predictions of deflections and failure points associated with the material’s weaker axis. However, the low anisotropic nature intrinsic from the printed orientation could be simplified with the use of an average and well-adjusted von Mises failure model with adequate security factor, which ensures proper results for the intended fixture design. Since these values have been obtained under strict experimental protocols, these mechanical properties of PLA will be used in this finite element model and in topological optimization. Previous studies have confirmed the significant strain rate dependency PLA, with its stress–strain curves exhibiting typical thermoplastic behavior where yield stress increases with higher strain rates, highlighting the material’s sensitivity. While quasistatic loading of PLA fixtures remains within the elastic domain where time and temperature-dependent effects are negligible, this behavior becomes a crucial design consideration for high-speed testing to prevent failure and ensure optimal performance [62]. On the other hand, the research by Valenzuela et al. [63] demonstrated that PLA can be used in the manufacture of rigid molds that are traditionally produced in steel, achieving adequate structural performance for demanding engineering applications. This finding shows that it is possible to replace metal structures with optimized PLA components, provided that the structural design is carefully developed. Criteria such as rigidity, structural symmetry, and ease of manufacture were considered in defining the geometry of the clamping system. Four successive iterations of the support design were carried out, each of which was modified based on the results obtained by finite element analysis (FEM), specifically considering the equivalent stress distribution (von Mises) and the total deformation. These variables allowed the identification of critical zones of stress concentration and unnecessary stiffness, which guided the decision-making for the geometry redesign in each iteration. In each iteration, new reinforcements were incorporated in the critical zones in order to increase the stiffness of the support and its safety factor. Figure 2 shows the iterative pre-design process, in which additional stiffeners were incorporated in each iteration with the aim of increasing the safety factor (SF) of the support. In the first design, an SF value of 2.08 was achieved, which was considered insufficient in view of the cyclic load requirements and possible stress concentrations, according to von Mises. Therefore, successive modifications were made to progressively increase the structural capacity until a final SF value of 4.25 was achieved. This threshold was defined as optimal, as it guarantees rigid support behavior to adequately transmit the cyclic load protocol to the Kelvin-type structure. In this way, the final design adequately balances structural rigidity, efficiency in the use of material, and feasibility of manufacturing through FFF.

**Figure 3:**
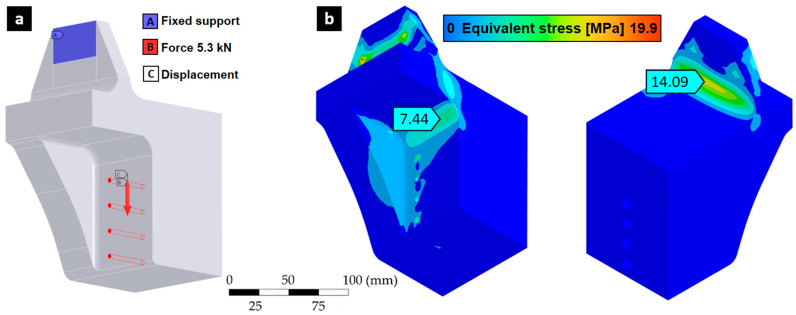
(**a**) Boundary conditions for the FEM model and (**b**) analysis results through the equivalent stress distribution according to von Mises.

This mechanical fixture design is specifically designed to enable axial cyclic testing of Kelvin lattice metal structures manufactured using laser powder bed fusion (LPBF). The design aims to ensure reliable load transfer, dimensional compatibility with the Instron 8801 universal testing machine, and ease of manufacture using fused filament fabrication (FFF) technology with PLA. The final geometry used for this procedure corresponds to the final design iteration shown in Figure 2. This meets the mechanical strength requirements, according to FEM analysis. It reached a maximum stress of 14.09 [MPa] according to the von Mises equivalent stress distribution, as shown in Figure 3.

The objective of stopping the iterative process until reaching an SF of 4.25 responds to a criterion of balance between structural performance and material optimization. Although it was possible to continue adding stiffeners to achieve even higher safety factors, this would have meant a significant increase in the mass of the support and in material consumption, which could reduce the efficiency of topological optimization. Furthermore, higher SF values do not necessarily translate into better performance under cyclic loads, as the system’s response is conditioned by the overall stiffness and the correct transmission of forces to the test specimen.

### 2.3. Topology Optimization Procedure

**Figure 4:**
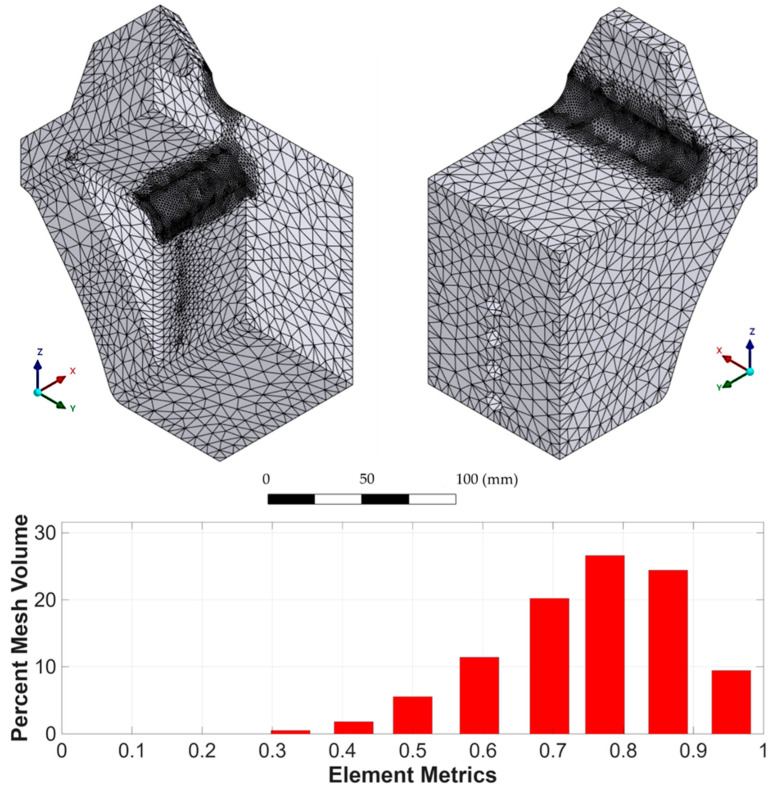
Mesh of the mechanical testing fixture device for topology optimization.

The three-dimensional (3D) topological optimization process was carried out with the aim of minimizing the mass of the mechanical testing fixture device, while preserving its mechanical rigidity under cyclic load conditions. The design is coupled with the geometry of the Kelvin-type structure, avoiding stress concentration and preserving visual access to critical areas during testing. The optimization was performed with ANSYS Mechanical using the density-based topology optimization method, which iteratively redistributes material within the design domain to minimize structural compliance (i.e., maximize stiffness). A mass retention constraint of 60% was imposed to ensure that the optimized geometry achieved a significant weight reduction while still maintaining adequate structural integrity. This constraint prevented excessive material removal that could compromise manufacturability or lead to premature failure. The optimization process also preserved the boundary regions in contact with the specimen and testing machine, ensuring proper load transfer and fixture functionality. By minimizing compliance under these constraints, the resulting designs achieved an optimal balance between lightweight fabrication and mechanical performance. The structural load and boundary conditions applied in the optimization involved applying an axial compressive load of 5.3 kN to the contact surface with the lattice specimen, while the clamping surfaces were completely constrained (Figure 3). The optimization domain included the entire fixture volume except for the surfaces where these boundary conditions were applied. The mesh used for the topology optimization is shown in Figure 4.

For structural optimization, PLA filament was considered as the material, which was modeled as linear elastic using the mechanical properties defined by the main printing direction. The accessory was printed with 100% infill, with alternating linear patterns rotated 90° between layers to maximize isotropy and mechanical strength. The design included interface zones for proper alignment within the testing machine clamps and a contact surface adapted to the base of the lattice sample. To account for the flexibility of the system introduced by the PLA fixture itself, a displacement correction method based on the technique proposed by Tuninetti et al. [64] was applied, which allows the actual mechanical response of the tested sample to be isolated by subtracting the deformation induced by the fixture. This correction allows for the validation of additively manufactured components under cyclic loading, which was also verified by dimensional analysis based on digital image correlation (DIC). The average correction factor was determined to be 6% of the total measured displacement, which was used to adjust the experimental data for further analysis. The performance of the accessory was also validated experimentally by means of a uniaxial tensile test on the complete accessory assembly under real boundary conditions. The results were compared with FEM simulations, confirming good agreement in terms of global deformation and confirming that the accessory remains within the elastic range throughout the loading protocol. A tetrahedral mesh was used in the design space, and sensitivity to convergence was addressed by refining in areas of high stress. The optimization algorithm generated a voxel-based geometry, which was then manually smoothed and reconstructed in Autodesk Fusion 360 to ensure printability and structural consistency.

The experimental validation of the optimized topology fixings will be carried out using uniaxial tensile tests under controlled load conditions. Two identical support components will be manufactured using fused filament fabrication (FFF) in PLA and assembled opposite each other, replicating the boundary conditions of the cyclic test configuration. The assembled fastener will be subjected to a monotonic tensile load using an Instron 8801 universal testing machine, with displacement and force data recorded in real time. The test will be performed under displacement control at a constant speed of 0.5 mm/s, and the resulting force–displacement curve will be compared with predictions obtained using finite element analysis (FEM). In addition, digital image analysis will be performed using high-resolution video frames and CAD-based measurement references to quantify the relative deformation of the fixture. This will allow the identification of any unwanted localized deformation in the support structure. The objective of this validation is to confirm that the optimized design remains within the elastic range under the anticipated maximum load and does not introduce significant artifacts in the mechanical response of the test sample.

## 3. Results and Discussion

### 3.1. Topology-Optimized PLA Fixtures for Cyclic Testing

**Figure 5:**
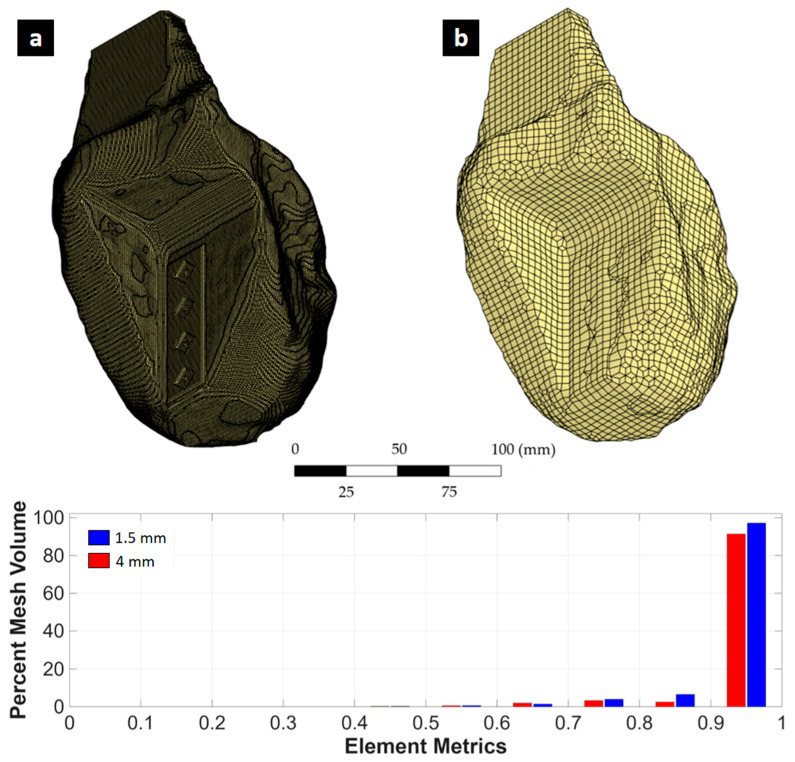
Comparison of the structure with topological optimization for two different meshes with different element sizes: (**a**) 1.5 [mm] and (**b**) 4 [mm].

The application of topological optimization to the mechanical device enabled the development of a new design that significantly improves structural efficiency while maintaining the stiffness requirements for cyclic load testing of cellular structures. The optimized device achieved a mass reduction of approximately 40% compared to the latest version shown in Figure 2, demonstrating the effectiveness of the optimization strategy in reducing material usage without compromising safety or stiffness. Figure 5 shows the geometry obtained after applying the topological optimization process to the cyclic test fixture. Successive mesh refinements showed that von Mises stress values changed by ~5.0%, 0.6%, and 5.3% when moving from 4 → 2 mm, 4 → 1.5 mm, and 2 → 1.5 mm meshes, respectively. For maximum deformation, the corresponding variations were ~3.0%, 6.9%, and 4.0%. These results confirm that beyond the 2 mm mesh size, the FEM outputs stabilize with variations below ~5%, indicating that the simulations are mesh-independent within acceptable numerical error margins. This geometry corresponds to a minimum mass solution compatible with the imposed boundary constraints and the expected load field during the test. Despite the resulting morphological complexity, the final geometry presents a continuous distribution of material in the critical load areas, with a clear elimination of unnecessary volumes. Table 1 summarizes the comparative values of the optimized geometry for different mesh element sizes. Notably, there is a 39.8% reduction in mass, while maximum deformation decreased by 12.4% and maximum equivalent stress by 9.2%, confirming that the resulting topology is not only more efficient in terms of material use but also improves the overall mechanical conditions of the component.

**Figure 6:**
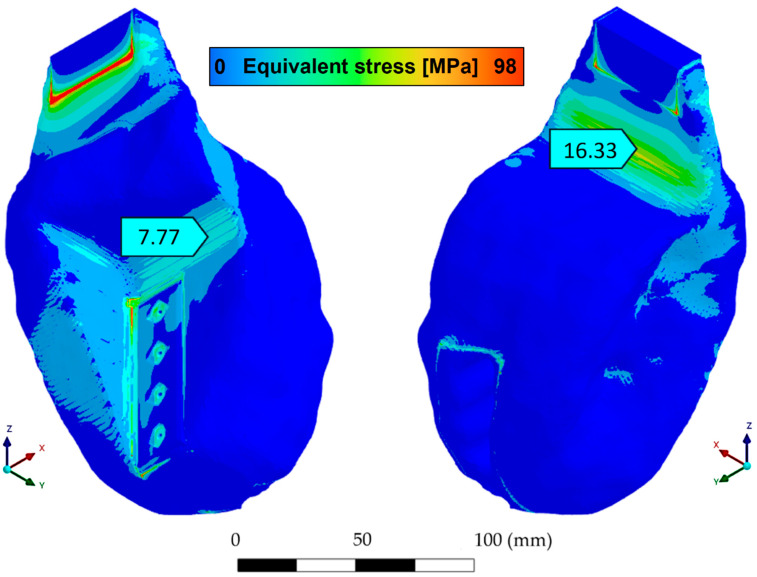
Equivalent von Mises stresses in optimized support with element size of 1.5 [mm].

On the other hand, Figure 6 shows the von Mises equivalent stress map for the optimized support. It can be seen that the areas of maximum stress are concentrated in the regions connecting the test specimen and the supports, with maximum values remaining around 16.33 [MPa], which represents an SF greater than 3.5, considering the ultimate tensile strength of PLA to be approximately 60 [MPa]. This magnitude confirms that the optimized support will operate completely within the elastic range even under the maximum load considered in the tests of 6 [kN]. These values validate the robustness of the device for use in cyclic testing protocols and indicate that the optimization process successfully maintained mechanical integrity while significantly reducing unnecessary material. One of the most valuable results of the optimization process is the redistribution of load paths within the structure. The topological optimization algorithm identified and retained key load-bearing regions while eliminating non-critical volumes. This resulted in an organic geometry that not only reduces weight but also improves stress flow and minimizes deformation under axial load. The smoothed voxel-based reconstruction of the output further ensured that the design could be efficiently manufactured using standard FFF technology, without the need for post-processing or support material.

### 3.2. Validation of Stiffness and Load Transfer

**Figure 7:**
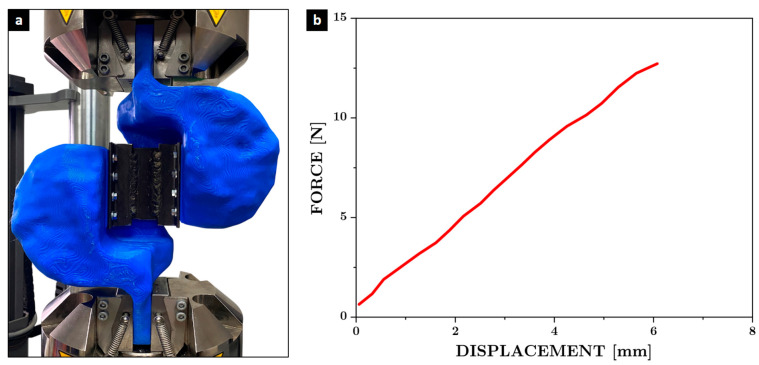
(**a**) Uniaxial tensile test of the fixture device made of PLA showing (**b**) the force–displacement curve.

The validation of the simulations was a multi-faceted process that combined two distinct experimental methods. Uniaxial tensile test and cyclic load test with digital image analysis. The purpose of the uniaxial tensile test was to confirm that the fixture’s supports exhibited linear, elastic behavior and did not undergo plastic deformation under load. The results showed a good agreement with FEM in terms of global deformation and stiffness, confirming that the accessory remains within the elastic range throughout the loading protocol and validating the accuracy of the numerical model used in the design and optimization stages. Regarding the cyclic load test, digital image analysis was used to measure the deformation of the optimized supports in real-world conditions. The analysis confirmed that the supports maintained their structural rigidity and experienced minimal deformations (an average of 0.73 mm), consistent with the simulations. In order to evaluate the rigidity of the optimized supports under real loads, the fastening devices were manufactured using an FFF system with PLA filament. A tensile test was carried out in which the deformation was recorded as a function of the applied force. The tensile test was conducted at a crosshead speed of 5 mm/min, following the recommendations of ASTM D638 [65] for rigid plastics in order to ensure quasistatic loading conditions. Although the testing procedure was based on this standard, it is important to note that the specimen was not a standardized dog-bone geometry, but rather a topology-optimized support specifically designed for this study. The objective of the test was not to obtain standardized material properties, but to analyze the deformation behavior of the component as a function of the applied load and to determine the maximum force it was able to withstand. To do this, the two supports were assembled facing each other using a rigid metal piece located in the joint area, in order to simulate the actual load conditions of the component during operation. As shown in Figure 7, a uniaxial tensile test was carried out to experimentally validate the rigidity of the optimized clamping system. To do this, two identical support devices were connected by bolts through a rigid A-36 steel connector, which ensures direct load transmission between both ends, without additional deformation interference. This configuration allows the mechanical behavior of the supports to be isolated, focusing exclusively on their response to axial load. The test was performed under displacement control, replicating the conditions expected for cyclic testing, and the force–displacement curve of the system was recorded. The results obtained show linear behavior in the elastic range, with no plastic deformation or instability phenomena, confirming that the supports operate within their safe stress range, considering that the maximum load for the cyclic load test will be 5.3 [kN]. In addition, the slope of the curve obtained in this assembly was consistent with the stiffness estimated in the finite element analyses, validating the accuracy of the numerical model used in the optimization stage. This controlled tensile test represents a key stage in the functional validation of the design, as it confirms that the optimized geometry does not introduce parasitic deformations or compromise axial alignment, which is essential to ensure the repeatability and accuracy of future cyclic tests on structural specimens. It is important to note that this study was limited to load levels of up to 5.3 kN. The performance of PLA fasteners under higher loads has not yet been experimentally verified, and it is expected that materials such as PETG, ABS, or fiber-reinforced filaments will need to be substituted to ensure structural integrity under more demanding conditions.

**Figure 8:**
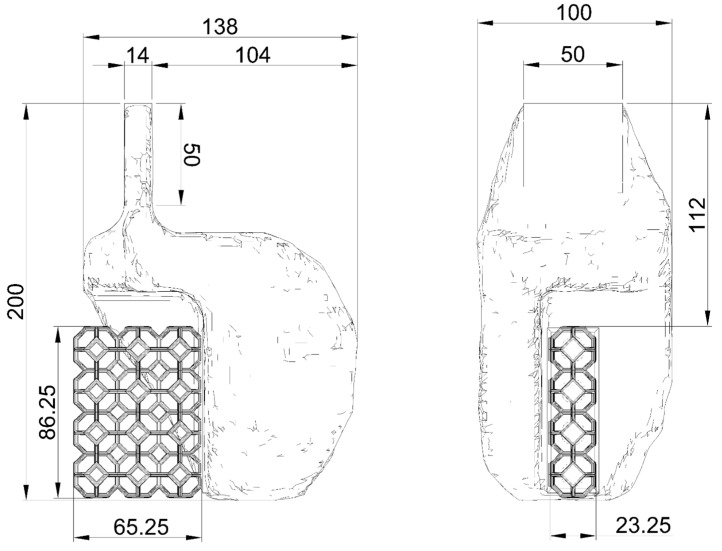
Sketch of the final design of the topologically optimized support. The drawing includes all relevant dimensions [mm] required for manufacturing.

Figure 8 illustrates the reconstructed model with smoothed surfaces based on the voxelized results of the optimizer. This final model was adapted for manufacturing using FFF, maintaining geometric fidelity with the original proposal and ensuring printability without additional support structures. This successful transition from the computational design space to the manufacturable physical object represents a highlight of this work. Where a notable aspect is the geometric simplicity of the final solution which preserved manufacturability despite the complexity of the optimization domain, by restricting the non-designed regions, specifically at the interfaces with the sample and the machine clamps, the final design maintained compatibility with the experimental setup and ensured repeatability under test conditions. By using algorithms to place material only where it is structurally required to handle specific loads, topology optimization creates highly efficient, lightweight structures. This approach removes the unnecessary mass common in conventional, over-engineered designs. The resulting organic shapes also enhance mechanical reliability by eliminating sharp corners that cause stress concentrations. By distributing forces more evenly, these optimized parts are stronger, more durable, and less prone to fatigue failure. This synergy produces fixtures with a superior strength-to-weight ratio, perfectly suited for additive manufacturing.

### 3.3. Cyclic Loading Tests on Kelvin Lattice Structures

**Figure 9:**
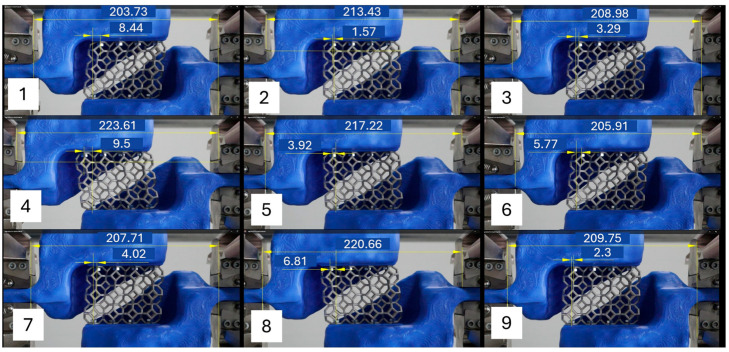
Graphical comparison of the sequential deformations (**1**–**9**) recorded in the test system: machine clamps, Kelvin-type structure, and optimized PLA supports; all measurements are in [mm].

In order to experimentally validate the effectiveness of the optimized supports, cyclic load tests were carried out on cellular structures with Kelvin topology. These tests are essential, as they allow us to evaluate not only the rigidity of the PLA-designed suction devices, but also the ability of the fastening system to ensure that the results obtained correspond exclusively to the intrinsic behavior of the unit cell. The choice of Kelvin topology is due to its wide application in studies of energy absorption and resistance under dynamic loads, making it a representative case study for validating the reliability of the support devices proposed in this work. Figure 9 presents a sequence of images arranged in a matrix format that illustrates the progressive deformations undergone by the Kelvin-type cellular structure during the cyclic loading test. These images correspond to different stages of the loading cycle, captured by means of recordings synchronized with the displacement imposed by the testing machine. In all the phases observed, it is evident that the supports manufactured in PLA using 3D printing and are topologically optimized maintain their structural rigidity and do not show appreciable deformations, which confirms their effectiveness as fastening systems. In addition, the displacement analysis shows that the transmission of forces between the test piece and the actuators occurs in a stable manner, without misalignments or loss of contact, which functionally validates the optimized design. Table 2 shows the displacements of the machine clamps, the deformation suffered by the Kelvin-type structure, and the deformation of the optimized supports, where it can be seen that the displacement of the jaws is transmitted almost entirely to the Kelvin-type structure. This behavior is particularly noteworthy considering that the supports were manufactured with a polymeric material, which highlights the geometric efficiency of the design in compensating for the mechanical limitations of the base material. In addition, the correct rigidity of the supports allows the deformations observed in the test specimen to correspond exclusively to the behavior of the Kelvin-type structure, without interference from the clamping system, thus guaranteeing the validity of the experimental data obtained. These results confirm that the topological optimization process not only reduced mass and material, but also delivered a highly functional, accurate, and reliable design for mechanical testing of complex structures.

Figure 9 allows us to visualize the deformations of the system at all stages of the cyclic load test. Table 2 compares the displacements for each component: the machine clamps, the Kelvin-type structure, and the optimized supports. In this way, it can be seen that the machine clamps experience displacements that are transmitted to the Kelvin-type structure through the optimized supports in PLA. The latter experience minimal deformations. This behavior confirms that the proposed design fulfills its main purpose: to rigidly transmit the forces to the specimen without absorbing significant deformations. These results are relevant because they demonstrate that optimized supports do not introduce additional flexibility into the system, preserving the validity of the cyclic test. Thus, the ability of a structural component to minimize relative deformation in response to external stresses is a critical factor in the design of test fixtures and clamping devices, as it ensures the correct representation of the phenomena under study. In this case, the topological design made it possible to obtain a lightweight geometry that maintains rigidity without compromising the integrity of the test, in line with what has been reported in studies on the optimization of test devices to replace steel components [63].

Furthermore, the low deformation recorded in the supports validates the decision to stop the iterative process at a safety factor of 4.25. This confirms that the final design offers an optimal balance between rigidity, material efficiency, and ease of manufacture, avoiding oversizing of the structure. Consequently, the results demonstrate the applicability of PLA as a raw material for this type of structural support in cyclic load tests. This work represents a significant advancement in the field of experimental mechanics, demonstrating for the first time that fixtures designed with FEM modeling and produced via FFF can yield adequate results for the cyclic testing of complex hysteretic lattice samples. We anticipate this methodology will serve as a foundational step, encouraging more focused research that promises to improve and expand the applications of our proposed methods. The primary significance of this research lies in its potential to democratize and accelerate the process of mechanical testing. Traditionally, researchers rely on expensive, custom-machined metallic fixtures that involve long lead times and high costs, creating a significant barrier to entry and slowing the pace of innovation. Our work challenges this paradigm by introducing a rapid, low-cost, and highly adaptable alternative.

This methodology will benefit the scientific community in several keyways. Cost and time reduction can be achieved by leveraging affordable FFF technology. Laboratories can drastically reduce the financial and time investment required for experimental setups. This allows for more extensive testing campaigns and faster iteration cycles. Additionally, it will create an enhanced design freedom for researchers, which are no longer limited by the constraints of traditional manufacturing. Fixtures can be precisely tailored to the unique and complex geometries of novel specimens, such as lattice structures, ensuring a more accurate application of loads and replication of boundary conditions. And finally, the ability to move from a digital design to a physical, test-ready fixture in a matter of hours allows for the rapid validation of new concepts. This agility is particularly beneficial for the development of architected materials and metamaterials.

This successful proof-of-concept opens a new line of inquiry focused on optimizing additively manufactured fixtures for a wider range of applications. We expect future work to explore the integration of more advanced computational tools, such as the topology optimization techniques used to intelligently distribute material and maximize strength-to-weight ratios [61]. Further research could also investigate the use of higher-performance composite filaments and expand this methodology to other challenging dynamic testing regimes, fundamentally changing how experimental validation is approached across multiple engineering disciplines.

It is important to note that the combination of topological optimization and additive manufacturing was essential to achieving the final design. While steel or other metals have traditionally been the most commonly used materials for this type of accessory, the optimized PLA supports demonstrated sufficient stiffness with minimal deformation during cyclic load testing. This confirms that 3D-printed accessories can effectively replace heavier and more expensive steel structures, offering flexibility for future design iterations. Three-dimensional printing offers several advantages for the development of advanced structures. It allows for the rapid creation of complex structures and easy design iteration, the manufacture of highly customized geometries, and reduced production costs in small batches. In addition, through 3D printing, it is possible to incorporate bolted joints with high mechanical strength into the design, as demonstrated in this study, without the need for post-machining.

These results highlight the effectiveness of the topologically optimized design of the PLA supports. On the one hand, the comparative analysis of deformations (Figure 9 and Table 2) shows that, under the applied cyclic loads, the lattice structure experienced deformations of up to 9.5 mm, while the machine recorded values of 11.65 mm. The optimized PLA supports exhibited considerably lower deformations, averaging 0.73 mm, which represents less than 8% of the deformation observed in the recorded machine values. This finding confirms that the supports act as rigid elements, capable of efficiently transmitting forces without compromising the stability of the system. Thus, the results obtained here demonstrate that topologically optimized PLA can satisfactorily fulfill the role of rigid support in cyclic load tests. Traditional support devices are usually made of steel or metal alloys, and the use of polymers in high-rigidity applications is rarely explored. This finding helps to fill a gap in existing research, in which most studies focus on the design of lattice structures [66,67] but not on the development of fastening systems adapted to this type of testing. Overall, the most important findings of this study can be summarized in three main points: (i) the numerical validation of a PLA support design capable of transmitting loads rigidly with negligible deformations, (ii) the demonstration that topological optimization can substantially increase the safety factor without penalizing material use, and finally (iii) the proposal of an alternative fixture to metal ones, which opens up new possibilities for lightweight, economical designs adapted to testing lattice materials under cyclic loads.

On the other hand, the selection of PLA for the fabrication of test fixtures represents an unconventional approach, departing from the high-stiffness metallic materials typically employed in mechanical testing. The primary motivation for this choice lies in the unparalleled design freedom and rapid, low-cost fabrication offered by FFF, which enabled the creation of custom grip geometries tailored precisely to the damper specimens. However, the inherent material limitations of PLA necessitated a carefully considered experimental methodology to ensure the fidelity of the results. The well-documented drawbacks of PLA include its relatively low stiffness, low strength, susceptibility to creep under sustained load, and a low glass transition temperature (Tg) [68]. In a conventional test setup, the low stiffness of the fixture would lead to significant elastic deformation under load. This fixture displacement would be erroneously captured by the testing machine’s crosshead displacement sensor, leading to a significant overestimation of the damper’s displacement and a distorted hysteretic loop, rendering the affected data [69]. PLA fixtures are limited by creep, fatigue, and temperature sensitivity, but these can be mitigated with material upgrade, hybrid design (PLA for general geometry and reinforcing critical regions with metal inserts or composite layers), and environmental control. The proposed optimization framework can indeed be extended, though it would require incorporating advanced fatigue, creep, and multiaxial material models into simulations and optimization criteria. To overcome this critical measurement challenge, a non-contact digital measuring was implemented. By tracking the displacement directly on the surface of the damper specimen, the method effectively decouples the measurement from the test frame and fixture. This approach ensures that the recorded force–displacement data corresponds exclusively to the damper’s performance, thereby nullifying the influence of fixture compliance on the final results. The success of this approach is contingent upon operating at low quasistatic strain rates and ensuring the test duration is in a range that prevents significant dimensional changes in the fixture due to creep [70]. Furthermore, the peak forces generated during the hysteretic cycles were carefully monitored to maintain a substantial factor of safety relative to PLA’s ultimate tensile strength. Thermal effects were considered negligible due to the low speed and short duration of the tests, which prevented significant heat generation in the damper.

In summary, while PLA fixtures are not a universal substitute for metallic fixtures in high-force or long-duration fatigue testing, our findings demonstrate their feasibility for testing hysteretic dampers. When combined with specimen-focused measurement techniques, additively manufactured PLA fixtures provide a valid, cost-effective, and highly adaptable alternative for specific, well-constrained testing scenarios. The optimization strategy implemented in this study demonstrates an appropriate balance between structural performance, material efficiency, and manufacturability. These results not only validate the accessory’s suitability for experimental testing of Kelvin-type networks but also demonstrate the broad potential of topological optimization for the design of auxiliary components in mechanical testing environments. Furthermore, the results obtained show that the topological optimization process implemented allowed for the design of a highly efficient structural support, significantly reducing the volume of material without negatively affecting the expected mechanical behavior. This simultaneous improvement in mass, stiffness, and strength represents an optimal solution from a structural and manufacturability standpoint. Similarly, the possibility of manufacturing the support using FFF printing without additional supports facilitates its implementation in low-cost, rapid iteration experimental environments, which is truly valuable for applied research and prototyping laboratories. In future work, this approach will be implemented for other test conditions or types of test specimens, including other lattice topologies or materials with nonlinear behavior. In addition, one could consider designing an optimization with different mass constraints to select the optimal value in terms of maximum flexibility, strength, and mass reduction. Although this study focused on Kelvin lattice structures printed in PLA, the proposed methodology is general and can be applied to other lattice geometries, such as Octet, Rhombic Dodecahedron, or auxetic designs, as well as to various materials, including other polymers, metal alloys, and composite systems. Therefore, the combination of design, additive manufacturing, and mechanical characterization can be extended to optimize different structural configurations and material properties for a wide range of engineering applications.

## 4. Conclusions

This research presents the design and topological optimization of supports for cyclic load testing, manufactured in PLA using 3D printing. Structural efficiency was maximized and material use was minimized, while maintaining the rigidity and functionality required for cyclic load testing of lattice structures. The results obtained demonstrate that the methodology implemented, which combined finite element simulations, geometric redesign iterations, and a topological optimization algorithm, achieved a mass reduction of nearly 40% compared to the base design, without compromising structural integrity. The simulations showed a safety factor greater than 3.4, confirming that the optimized support remains within the elastic range under the maximum load of 5.3 [KN].

In addition, preliminary experimental analysis using tensile testing through a steel connector showed that the supports respond in a rigid and stable manner, validating the predictions obtained through FEM. The combination of rigidity, low deformation, and adequate stress distribution demonstrates that the applied topological optimization was effective and can be a robust tool for the design of clamping devices in mechanical testing.

Furthermore, numerical simulations confirmed that the optimized supports exhibit minimal deformation under load, maintaining their structural integrity under cyclic conditions. This behavior was evidenced through digital image analysis, where it was determined that the displacements in the supports were significantly lower than those in the machine clamps and the Kelvin-type structure itself. This performance ensures the reliability of the tests by avoiding interference or loss of stiffness during load transmission.

Together, these findings confirm that it is possible to replace traditional metal structures with optimized PLA components, provided that the design is supported by rigorous numerical analysis and experimental validation protocols. Finally, this work opens up the possibility of extending the proposed methodology to other test devices and engineering applications where stiffness and precision are determining factors.

## Figures and Tables

**Table 1:** Comparison of the main results for optimized support using mesh convergence.

Element Size [mm]	Nodes	Elements	Equivalent Stress von Mises [MPa]	Maximum Deformation [mm]	Safety Factor [SF]	Mass [kg]
5 *	133,830	90,695	14.09	0.395	4.25	1.809
4	64,933	13,927	16.43	0.505	3.65	1.06
2	480,166	110,949	17.25	0.49	3.48	1.06
1.5	3,743,812	897,988	16.33	0.47	3.67	1.06

(*) non-optimized design as reference.

**Table 2:** Comparison of the displacements of the machine clamps, the lattice structure, and the optimized supports during the cyclic load test.

Step	Machine Jaws Displacement [mm]	Sample Deformation [mm]	Deformation of Optimized Supports [mm]
1	8.22	8.44	0.22
2	1.48	1.57	0.09
3	2.98	3.29	0.31
4	11.65	9.50	2.15
5	5.27	3.92	1.35
6	6.05	5.78	0.27
7	4.24	4.02	0.22
8	8.70	6.81	1.89
9	2.21	2.30	0.09
		Average	0.73

## Data Availability

The original contributions presented in this study are included in the article. Further inquiries can be directed to the corresponding authors.
